# Epigenetic Effects of Drugs of Abuse

**DOI:** 10.3390/ijerph15102098

**Published:** 2018-09-25

**Authors:** Thomas Heinbockel, Antonei B. Csoka

**Affiliations:** Department of Anatomy, Howard University College of Medicine, Washington, DC 20059, USA; antonei.csoka@Howard.edu

**Keywords:** brain, cannabinoid, central nervous system, drug addiction, gene-environment interaction, gene expression, health disparity, marijuana, neuroscience

## Abstract

Drug addiction affects a large extent of young people and disadvantaged populations. Drugs of abuse impede brain circuits or affect the functionality of brain circuits and interfere with bodily functions. Cannabinoids (Δ9-tetrahydrocannabinol) form key constituents of marijuana derived from the cannabis plant. Marijuana is a frequently used illegal drug in the USA. Here, we review the effects of cannabinoids at the epigenetic level and the potential role of these epigenetic effects in health and disease. Epigenetics is the study of alterations in gene expression that are transmitted across generations and take place without an alteration in DNA sequence, but are due to modulation of chromatin associated factors by environmental effects. Epigenetics is now known to offer an extra mechanism of control over transcription and how genes are expressed. Insights from research at the genetic and epigenetic level potentially provide venues that allow the translation of the biology of abused drugs to new means of how to treat marijuana substance use disorder or other addictions using pharmacotherapeutic tools.

## 1. Introduction

Drugs of abuse are drugs that are taken for non-medicinal reasons. They impede brain circuits or affect the functionality of brain circuits and interfere with bodily functions. Drug addiction affects a large extent of young people and disadvantaged populations. Here, we review how a family of abuse drugs, cannabinoids, acts at the epigenetic level and the importance of these epigenetic changes in addiction. The aim of research on this topic is to understand the genetic mechanisms of such addiction. It provides potential venues that allow the translation of the biology of abused drugs to new treatment options of marijuana substance use disorder using pharmacotherapeutic tools. Research will help to develop effective prevention of the consequences of using drugs of abuse and therapeutic strategies that may eliminate the impact of drug abuse in terms of altered gene expression and contribute to a reduction of health disparities. Health disparities are differences in health outcomes that have been found in disadvantaged populations [1,2]. These include increased frequency and/or occurrence of a disease, early and/or disproportionate death and subordinate health-related life quality [1,2]. Modern research aims to advance scientific knowledge about the defining mechanisms of how health factors affect disparities and how we can generate knowledge that is translated into interventions to reduce disparities. The mechanisms that lead to health disparities include (a) individual behaviors, lifestyle, beliefs and response to stress; (b) physical and cultural environment; (c) clinical events and health care; and (d) biological processes, genetics and epigenetics. This can result in the premature beginning of a disease, rapid development of diseases or increased vulnerability.

## 2. Epigenetics as a New Level of Modulation

In order to understand mechanisms of development, physiology and evolution, the genome of humans and selected other organisms has been sequenced [3,4,5]. More recently, a second level of genetics has been described, termed epigenetics. Here, chemical modifications of the DNA are studied as well as interactions that include genome associated proteins. It analyzes differences in the expression of genes that are heritable and arise without a change of the DNA sequence. Instead, they are due to modulation of chromatin associated factors by environmental effects. Epigenetic mechanisms afford another mechanism of transcriptional control in regulating gene expression. Evidence shows that epigenetic modifications are linked to changes in development and behavior as well as with genetic disorders and various diseases [6]. It is now known that, as human cells age, widespread epigenetic changes occur across the entire genome [7]. This is commonly known as the “epigenetic clock” phenomenon. Recently, it has been discovered that there is also ethnic variability in this epigenetic clock [8]. Interestingly, a comparison of extrinsic epigenetic aging revealed that the rates in African-Americans are lower than in Caucasians and Hispanics.

## 3. Synthetic Cannabinoids

The endogenous cannabinoid system critically involves a plant-derived compound, namely, Δ9-tetrahydrocannabinol, THC, which is the main psychoactive substance in cannabis plants and the bioactive ingredient of the drugs marijuana and hashish. The endocannabinoid system (cannabinoid receptors and their ligands cannabinoids) has been shown to act as a neuromodulator in the healthy brain [9,10]. Cannabinoids are hypothesized to have the potential to be addictive in those systems of the brain that are related to motivation and stress. This is based on strong adaptations within the system through signal transduction mechanisms and also between systems based on alterations in the neural circuitry [11].

A new wave of drug abuse has hit many communities and impacts the clinical enterprise [12]. The new abused drugs are synthetic cannabinoids (SCs) that are thought to mimic classic marijuana, even though the clinical presentation of patients suggests that these ‘herbal products’ are cocktails of various stimulants, some of which are unrelated to cannabinoids and do not act through cannabinoid receptors. SCs comprise different psychoactive chemicals or a cocktail thereof. These chemicals can be applied by spraying them onto plant material to combine them with marijuana leaves. This is subsequently smoked or consumed to reach a ‘high’. These products are traded under a multiplicity of names (e.g., synthetic marijuana, K2, bizarre, spice, space cadets, black mamba, and crazy clown) and are retailed in stores as herbal products. The effects of these drugs are much more potent than THC and can lead to violent outbursts in hospital emergency rooms. Compared to THC, the majority of SCs show stronger binding affinity to the cannabinoid CB1 receptor. THC is a partial agonist at CB1. SCs have a stronger affinity at CB1 than CB2. Studies have demonstrated a pharmacological effect of SCs that is two to one hundred times more potent than that of THC. These effects include analgesic, anti-seizure, weight-loss, anti-inflammatory, and anti-cancer growth effects [12,13]. Contrary to THC, SCs can result in significant problems for the well-being, such as psychosis, hallucinations, anxiety, tachycardia, and violent behavior [14]. Other commonly reported SC toxicities are hypertension, delusions, agitation and irritability, vertigo, dizziness, drowsiness, nausea, confusion, chest pain and acute kidney injury [12,15,16]. Part of the popularity of SCs is resulting from complications in their detection by standard cannabinoid screening tests, which makes clinical identification of SC users difficult. The clinical presentations seen in hospitals are reflected by reports from the Center for Disease Control [17] which on 6 April 2015 received warning of an increase in telephone calls to U.S. poison centers in relation to SC use. Based on data followed by the National Poison Data System, SC use was up by 330% from 349 in January 2015 to 1501 in April 2015. While law enforcement agencies were able to actively control some of these new drugs, the producers of SCs often vary the chemical formulas to evade discovery and control [15]. Globally, the ingestion of synthetic mind-altering drugs, the “new psychoactive substances”, is developing tremendously [14,18]. SCs constitute the most widely taken new psychoactive substances, usually purchased as marijuana-like drugs, and thought of as risk-free drugs by the inexperienced user. The molecular interactions of these novel synthetic drugs that lead to altered cellular functions are obscure and immediate high-paced research is an utmost necessity to identify their cellular targets and, subsequently, to enhance clinical help to treat this new wave of patients [19,20]. 

Experiments to address the cellular effects of spice and other cannabimimetic drugs are just starting in animal models [21,22], whereas their long-term effects and genetic modifications are almost unknown. Previous work has already tested the effects of specific SCs that are available for research studies on brain cells such as olfactory neurons [10,23]. Marijuana (cannabis) is already the most widely abused illegal drug in the U.S. The use is now increasing even more with newly developed SCs. THC activates cannabinoid receptors (CB1R) in the brain just like endogenous cannabinoids (endocannabinoids, eCBs) that are expressed by nerve cells in the brain. eCBs are important neuromodulators [24,25,26,27,28,29,30]. Many of the actions of eCBs occur in an epigenetic context and are susceptible to experiences and exposure to factors in early life. This could be a starting point for understanding drug addiction and the nature of brain impairments that lead to neuropsychiatric conditions [31,32].

## 4. Addiction as a Biological Disorder

Studies of the brain have shown that addiction is a biological disorder [33]. Neurobiological studies of addiction mechanisms have revealed mechanisms of brain function, specifically with regard to reward, motivation, and emotions. The main feature of drug addiction is the chronical relapse of the drug habit. This is different from infrequent, controlled, or social drug use. As described by Koob et al. [11], drug addiction is described by several features: (a) compulsory drug seeking and taking; (b) lack of control of drug taking; and (c) appearance of an adverse emotional state such as dysphoria, anxiety, and irritability. If access to drugs is prevented a motivational withdrawal syndrome is apparent. Various studies have shown that addictions exhibit features of other disorders with respect to the chronic relapsing nature and the efficiency of treatments, namely diabetes, asthma, and hypertension [34].

The beginning of drug abuse (substance use disorder) is often related to social and environmental factors. In contrast, advancement to addiction is linked to changes in the brain. Addictive drugs modify neural networks and these modifications can persist beyond the time of drug intake. As pointed out by Koob et al. [11], for cannabis, 13.9% of last-year users fulfilled the criteria for addiction.

Drugs of abuse such as THC, a natural CB1 receptor agonist, can result in long-lasting addiction because of their modifications of endogenous neural pathways. Cannabinoid CB1 receptor antagonists offer an opportunity to serve as blockers of the effects of THC. THC, nicotine and ethanol have in common a direct reinforcing effect. Blockers of CB1 receptors can avert a relapse in drug use. This has been shown to be the case for cocaine, methamphetamine, and heroin [35]. The idea is supported by several studies that suggest a receptor blockade by CB1 receptor ligands could be an innovative strategy for drug users and be operative for various types of drugs.

The critical gap in our understanding of drug abuse is the physiological mechanisms that are in place and facilitate the switch from recreational and occasional drug use to subsequent drug addiction [11]. Drug addiction is linked to changes in the brain. Research needs to unravel the brain systems and genetic mechanisms of marijuana substance use disorder or, more generally, drug addiction, and develop pharmacotherapeutic treatment strategies that prevent drug abuse and eliminate health disparities. Therefore, the translation of lab results into better health among health disparity populations continues to be the main goal. Diseases with increased mortality and disease burden among health disparity populations requires study and will disclose gene-environment interactions and epigenetic mechanisms of health disparities. It has been estimated that about 40% of phenotypic variations in addiction is attributed to genetics [11]. This variability can be based on intricate genetic alterations including alleles involved in the control of drug metabolism or genes that confer drug sensitivity and input from the environment [11]. Heritability cannot account for all of the variability. This observation makes the case that gene–environment interactions are at play, for example specific stages of the addiction cycle, developmental factors, and social factors. Insights from such studies will afford us with a better understanding of drug addiction and provide us with new pharmacological treatment strategies to prevent the use of drugs.

## 5. Cannabinoid-Induced Epigenetic Changes

Little is known about how the epigenome and its changes are related to cannabinoids as discussed below. One mechanism of plasticity in the nervous system is based on a change in receptor expression, even though this is not an epigenetic phenomenon. For example, insertion or elimination of an ionotropic glutamate receptor, the α-amino-3-hydroxy-5-methyl-4-isoxazolepropionic acid (AMPA) receptor, results in modulating the strength of synaptic contacts in neural circuits [36,37]. This regulation has been attributed to the strength and coincidence of synaptic input to a given nerve cell. Synaptic transmission and plasticity in the hippocampus are affected by synthetic cannabinoids such as spice [21]. Work on human SH SY5Y neuroblastoma cells and human Jurkat T lymphocytes has demonstrated that cannabinoid receptors (CB1) and opioid (μ-opioid receptors) are subject to epigenetic regulation of their expression [38]. The induction of these receptor types by modifiers of the epigenome in specific cell types of the nervous and immune system can potentially increase the impact of the related drugs on neuronal and immune functions. From a translational point of view, this regulation could be helpful in the design of novel treatments for several diseases, e.g., multiple sclerosis. In this case, an increase of communication of cannabinoid or opioid signals is advantageous [38].

Along the same lines, another study addressed THC-induced epigenetic changes in immune cells [39] since THC has been demonstrated to relay powerful immunosuppressive and anti-inflammatory properties. The authors examined how THC affects global histone methylation in lymph node cells of mice. Their results indicated that THC triggers the expression of some genes while reducing the expression of other genes through histone modification. Functionally, the histone marker-associated genes had involvement in many cellular functions ranging from regulation of the cell cycle to metabolic functions. In the immune cells tested, the authors concluded that a pleiotropic effect on gene expression was at work in response to THC. THC modulated immune responses through epigenetic regulation that involved histone modifications. In addition to the immediate influence on gene expression in immune cells, cannabinoids were found to have long-term consequences and transgenerational effects on the immune system [40]. Furthermore, they exerted negative effects on the immune system in the developing fetus during prenatal exposure such as T cell dysfunction and lower immune response to viral antigens. The immunosuppressive properties of cannabinoids are based on epigenetic mechanisms. These include altered microRNA, DNA methylation and histone modification profiles. The results make an important statement, namely exposure to cannabis from parents or prenatal exposure can evoked epigenetic changes with consequences for the children’s immune system and additional transgenerational consequences [40].

Similar lasting consequences of exposure to cannabinoids have been observed in the nervous system, specifically in the nucleus accumbens. This brain structure is involved in reward processing [41]. The authors previously demonstrated that THC exposure modifies reward-related behavior and gene regulation in the F1 offspring generation that was not exposed to THC. The authors showed that F1 adult rats with parental THC exposure show electrophysiological impairments related to dysregulation of synaptic plasticity [42]. They have subsequently interrogated the epigenome of the nucleus accumbens and showed drug-related cross-generational epigenetic effects [41]. More specifically, the authors characterized systems-level changes in DNA methylation and were able to identify more than a thousand differentially methylated regions (DMRs) associated with parental THC exposure in F1 adults. Their results showed that many DMRs overlapped genes encoding regulators of synaptic plasticity, including glutamate receptors and kainate receptors (*Grin2a*, *Grik3*, and *Grik5*), G-protein-coupled receptors (GPCRs; *Gpr39*, *Gpr157*, and *Gpr158*), pre- and postsynaptic ion channels (*Cacna1a*, *Kcna5*, *Kcnma1*, *Kcnq2*, *Kcnh1*, *Kcnn1*, *Kcnm*, *Kcnj10*, *Kcnn4*, *Kcnq1*, *Hcn3*, *Scn5a*, and *Scn8a*) and scaffolding proteins. Through their work, the authors identified a network of DMR-associated genes that are involved in glutamatergic synaptic regulation. The genes showed modified mRNA expression in the nucleus accumbens. Variation in DNA methylation is one mechanism of epigenetic modification. It is known to be linked to environmental influences and often persists through multiple generations as discussed by Watson et al. [41]. Another study addressed epigenetic changes in the nucleus accumbens and also in hippocampus and amygdala, two key brain areas for spatial memory and emotions, respectively [43]. During development, the brain is particularly vulnerable to drug exposure, which can have lasting effects and result in mental health disorders. The authors studied the molecular basis for this adolescent vulnerability and confirmed that histone modifications are important epigenetic mechanisms after THC exposure, which resulted primarily in transcriptional repression in adolescent rats in contrast to transcriptional activation in the adult rat. As mentioned above, histone modifications include various types of posttranslational modifications. The most frequently studied histone modifications include acetylation and methylation, which have very different or even opposite effects on transcriptional activity [43]. Histone acetylation results in transcriptional activation; in contrast, histone methylation can lead to either transcriptional activation or repression, which depends on the methylation site. As described by Prini et al., on histone H3, methylation of K27 and K9 results in repression while methylation on K4 activates transcription [43]. The authors determined histone modifications associated with transcriptional repression (H3K9 di- and tri-methylation, H3K27 tri-methylation) and activation (H3K9 and H3K14 acetylation) after adolescent and adult chronic THC exposure. Their results demonstrate that chronic exposure to increasing doses of THC affected histone modifications in a region- and age-specific manner [43]. In the adolescent brain, the authors found primarily changes leading to transcriptional repression. In the adult brain, chronic THC exposure resulted in transcriptional activation.

## 6. Conclusions

Epigenetic mechanisms are increasingly considered as key factors in the development of psychiatric diseases. These epigenetic mechanisms act in specific brain-regions and at specific ages [43]. Overall, the data point to disturbances of the epigenome that are responsible for long-lasting, often tissue-specific, transcriptional and behavioral effects of cannabinoids and effects that cross generations [20,44]. The results reveal effects of environmental toxicological factors, namely drugs, or pharmacological agents (drug inhibitors) on gene expression, i.e., the epigenome during disease pathology. Therefore, these studies allow the determination of gene–environment interactions and can facilitate our knowledge gain of epigenetic mechanisms in health disparities. 
